# A Three-Dimensional Microstructure Reconstruction Framework for Permeable Pavement Analysis Based on 3D-IWGAN with Enhanced Gradient Penalty

**DOI:** 10.3390/s21113603

**Published:** 2021-05-21

**Authors:** Ludia Eka Feri, Jaehun Ahn, Shahrullohon Lutfillohonov, Joonho Kwon

**Affiliations:** 1 Department of Big Data, Pusan National University, Busan 46241, Korea; ludia.ef@gmail.com; 2 Department of Civil and Environmental Engineering, Pusan National University, Busan 46241, Korea; jahn@pusan.ac.kr; 3 School of Computer Science and Engineering, Pusan National University, Busan 46241, Korea; shahrullo@pusan.ac.kr

**Keywords:** 3D microstructure reconstruction, permeable pavement, deep learning, generative adversarial networks

## Abstract

Owing to the increasing use of permeable pavement, there is a growing need for studies that can improve its design and durability. One of the most important factors that can reduce the functionality of permeable pavement is the clogging issue. Field experiments for investigating the clogging potential are relatively expensive owing to the high-cost testing equipment and materials. Moreover, a lot of time is required for conducting real physical experiments to obtain physical properties for permeable pavement. In this paper, to overcome these limitations, we propose a three-dimensional microstructure reconstruction framework based on 3D-IDWGAN with an enhanced gradient penalty, which is an image-based computational system for clogging analysis in permeable pavement. Our proposed system first takes a two-dimensional image as an input and extracts latent features from the 2D image. Then, it generates a 3D microstructure image through the generative adversarial network part of our model with the enhanced gradient penalty. For checking the effectiveness of our system, we utilize the reconstructed 3D image combined with the numerical method for pavement microstructure analysis. Our results show improvements in the three-dimensional image generation of the microstructure, compared with other generative adversarial network methods, and the values of physical properties extracted from our model are similar to those obtained via real pavement samples.

## 1. Introduction

In recent years, permeable pavement has been widely used in developed countries as part of low-impact development practices [1]. The main benefit of permeable pavement is that it can allow rainwater to pass through its pores into the ground below by filtering rainwater over the distinct layers, thereby reducing the excessive volume of waterlogging. The runoff volumes and discharge rates can be reduced from paved surfaces and the high risk of downstream flooding can be decreased. Additionally, permeable pavement contributes water quality enhancement. It can trap stormwater pollutants and prevent them from reaching downstream receiving waters [2]. Other benefits of using this special pavement are skid resistance, noise control, and surface temperature reduction [3].

However, there are some drawbacks to using permeable pavement. First, it is expensive owing to its special design. Permeable pavement consists of aggregate components with different materials such as concrete mixtures with cement, polymer, and plastic [4]. Second, it has a high maintenance cost, especially for cleaning the pavement. An industrial vacuum is needed to remove the particles that can block the spaces in the pavement. If it is not maintained properly, the water and pollutants can run off the surface, which can cause flooding. Finally, it is prone to the clogging problem that is closely related to the maintenance issue. Clogging is the reduction in porosity and permeability when fine particles, sand, or clay block the spaces or pores between the pavers. The quality of permeable pavement can be measured using the clogging potential value. Clogging potential is the ratio of porosity or permeability reduction because of clogging to the initial porosity or permeability in the unclogged state [5].

Several experimental studies related to clogging in permeable pavement have been reported by researchers over the years to develop an ideal pavement design [6,7,8,9]. A good permeable pavement should have a small clogging potential value. According to the researchers, we need to understand the effects of different pore structures to yield a permeable pavement that is less susceptible to clogging [5].

To analyze the clogging potential in permeable pavements, some important properties of pavement microstructure need to be measured. Microstructure is the small-scale structure of a material that can be viewed through a microscope. The properties of microstructure are very important for understanding the material structure and studying the mechanical behavior of a material. In permeable pavement, three crucial properties for analysis are (1) porosity, (2) permeability, and (3) hydraulic conductivity [5,10]. Porosity is a fraction of the volume of pores over the total volume of a material. It is considered as a most important property since it affects the flow rates in a permeable pavement. Permeability is defined as the ability of a porous material to transmit fluids, and hydraulic conductivity is the ease with which a fluid (water) can move through porous surfaces [11]. Along with these properties, some morphological aspects also need to be carefully measured to study the clogging phenomenon in a porous medium like the permeable pavement [12].

Traditionally, to measure the clogging potential properties, we needed to conduct field experiments [6,13] using real physical materials. These are high in cost because the experiments require several samples and instruments that must be installed in the real field. The setting of a test area is another aspect that can be expensive, especially for conducting a large-scale field experiment. It is also time consuming, especially in large-scale experiments, owing to the equipment setup, material selection, and test-area preparation, which involves an intricate process. Thus, a more efficient method to measure the clogging potential properties is needed. Alternatively, they can be measured through visual experiments with image analysis and computer simulation.

A virtual experiment is another alternative to estimate the clogging potential properties [14,15]. It is more efficient in comparison to field experiments because it uses fewer samples and equipment for the experiment, and the required time is also not high as required in a field experiment. For this type of experiment, the most important aspect is the preparation of a good three-dimensional model for computer simulation. Before the upsurge of deep learning in image processing, researchers used statistical methods and some basic machine learning techniques such as support vector machines and the genetic algorithm [16,17] to reconstruct a 3D microstructure. Another method uses stochastic models such as a Gaussian random field [18] combined with hybrid optimization that generates a 3D porous material structure using a two-point correlation function and a cluster correlation function. The most common method for 3D reconstruction is the two-point correlation method [19,20,21]. It characterizes the microstructure based on certain statistical features and then performs an optimization process to build a 3D structure that matches those statistical features.

However, there are some limitations to the basic machine learning techniques and statistical approach. They require predefined knowledge of the materials [19], many different samples, and the generated model sometimes does not resemble the real sample. In the statistical case, there is a possibility that two materials with different properties may share first- and second-order statistical information and chord-distribution functions. Therefore, it has failed to yield good estimates of the macroscopic properties [20]. In brief, previous approaches did not yield realistic 3D microstructure reconstruction due to lack of (1) many different samples and (2) a good generative model.

To overcome these limitations, we propose a deep-learning-based 3D microstructure reconstruction framework for permeable pavement analysis as a virtual experiment method. Our system first takes a two-dimensional image to extract the latent features of the image. Then, it generates the corresponding 3D microstructure image that can be obtained through the concept of the generative adversarial network [22,23]. Since it is hard to achieve stability in the original generative adversarial network during the training process, we decided to implement a 3D improved Wasserstein generative adversarial network (3D-IWGAN) [24,25] with an enhanced gradient penalty. One of the main advantages of utilizing this generative adversarial network is that it can produce sharper, even degenerate distributions, while other 3D GAN-based methods show instability in generated results. Thus, compared to other methods [22], our framework achieves high profit from the enhanced gradient penalty and keeps the stability during the training process.

The main contributions of this paper are summarized as follows:

- We propose a deep-learning-based 3D microstructure reconstruction framework for permeable pavement analysis. Our proposed framework can effectively reconstruct a 3D microstructure image after taking only one 2D image. For this purpose, our framework applies several image preprocessing techniques to the input 2D images and extracts the latent features from them via the variational autoencoder (VAE) method.
- In comparison to other generative adversarial network methods, we obtained more realistic 3D microstructure images of porous pavements and stable outputs. This stability is the main result of using 3D-IWGAN with an enhanced gradient penalty.
- The accuracy of our results has been verified by evaluating some physical properties extracted from the generated 3D samples. On observing nine different output samples, the average error difference in hydraulic computation was found to be less than 5%.

The remainder of this paper is organized as follows. Section 2 provides a brief overview of related research work. Section 3 presents some preliminaries of GANs. In Section 4, the 3D model generation and analysis of the generated model are explained in detail. The experiment and evaluation of the experimental results are discussed in Section 5. Finally, the conclusion of our work and future work are presented in Section 6.

## 2. Related Work

In this section, we provide a brief review of the related work. There are three categories to be discussed: (1) microstructure reconstruction approaches, (2) general 2D/3D object reconstruction approaches, and (3) deep-learning-based microstructure reconstruction approaches.

### 2.1. Microstructure Reconstruction

Microstructure is the small-scale structure of a material that can be viewed through a microscope. In the fields of materials science and civil engineering, microstructure properties are very important to understand the structure of a material and study its mechanical behavior.

The most common and earlier work for 3D microstructure reconstruction uses the two-point correlation method [19,20,21]. It characterizes the microstructure based on certain statistical features and then performs an optimization process to build a 3D structure that matches those statistical features. After this, statistical methods and basic machine learning algorithms were applied for reconstruction of 3D microstructure. Some of the well-known machine learning methods include support vector machines (SVMs) [16] and the genetic algorithm [17]. Among others, stochastic methods have shown better results for the given tasks. For example, Jiang et al. [18] proposed a method that exploits a Gaussian random field with the combination of hybrid optimization. It uses a two-point correlation function and a cluster correlation function to generate a 3D porous material structure.

### 2.2. General 2D/3D Object Reconstruction

After the successful results shown by convolutional neural networks (CNNs) in the field of image classification and computer vision, researchers started considering using CNNs and other deep learning methods in their research on permeable pavement.

Since the core functionality of GAN enables us to generate a set of realistic (2D) images from the given sample, thus it is extended to generate 3D images (objects) [26]. Although GAN can accurately visualize the results, it suffers from a major drawback: the instability of GAN training. To guarantee the stability of GAN training, researchers proposed to leverage the Wasserstein distance (WGAN) [27] and to further improve training of the WGAN (IWGAN) [24].

Table 1 lists the different methods for object reconstruction (microstructure and non-microstructure). It shows that the deep learning method is more common in 2D object reconstruction than 3D object reconstruction. More specifically, there is still no application of GAN in pavement microstructure. Therefore, we attempt to fill this gap by designing a GAN for this case.

### 2.3. Deep-Learning-Based Microstructure Reconstruction

There were a few research works on deep learning applications for microstructure reconstruction before the GAN technique. One example of such applications is a research work by Cang, Ruijin, et al. [28]. It proposed a convolutional deep belief network (CDBN) to reconstruct heterogeneous materials. Recently, Tran and Tran [29] introduced a 2D microstructure reconstruction framework based on the image inpainting method to solve the microstructure reconstruction problem in three different contexts.

Owing to the proliferation of GAN, several research approaches exploit the generative power of GAN. Since GAN is used for microstructure reconstruction [30], it suffers from the intrinsic instability issue. We published the preliminary results of a deep-learning-based microstructure reconstruction using IWGAN [31].

Recently, more complex methods have been combined with GAN-based methods [32,33,34]. An end-to-end three-dimensional reconstruction framework of porous media from a single two-dimensional dataset has been proposed [32]. Shams et al. [33] introduces coupled generative adversarial and autoencoder neural networks to overcome instability for the reconstruction of realizations of three-dimensional data. The model gains efficient results by applying a gradient-descent-based optimization method for training and stabilizing the neural networks. A transfer learning technique [34] is exploited to integrate statistical descriptors with feature maps from a pre-trained deep neural network into an overall loss function for an optimization-based reconstruction procedure.

Our proposed system is different from the aforementioned recent approaches in the following aspects. First, we focus on reconstructing the 3D microstructure of permeable pavements and obtaining 3D images with a given 2D image. In this regard, an end-to-end framework [32] has the same goal as our system. However, our system exploits the characteristics of 3D-IWGAN, whereas an end-to-end framework uses the modified version of BicycleGAN [35], which is mainly for 2D-to-2D translation. Second, we provide the more detailed steps for pre-processing and the pavement analysis methods for checking the effectiveness of generated 3D images.

## 3. Preliminaries

### 3.1. GAN

The generative adversarial network (GAN) is a deep learning method that learns the representation of high-dimensional probability from a given dataset, and was introduced by Ian Goodfellow et al. [22]. For image microstructure reconstruction, the dataset is a set of training sample images of the probability distribution underlying the image space.

**Figure 1:**
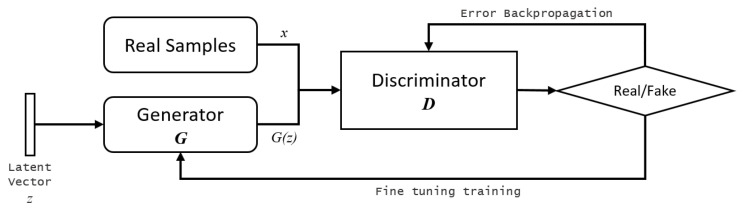
Generative adversarial networks. The discriminator network tries to distinguish between real and fake images, while the generator network tries to fool the discriminator by generating real-looking images.

GAN is a generative system that comprises two networks, a generator network and a discriminator network. The generator network converts latent vectors with a normal distribution into samples that can be classified as either real or fake by the discriminator network. The training process is stopped and the discriminator is discarded after a proper sample image is obtained from the generator. GAN follows a minmax game with a loss function that is formally defined as

(1) $$\min_{G}\max_{D}\left\{E_{x∼p_{data}}\left[logD\left(x\right)\right]+E_{z∼p_{noise}}\left[log\left(1-D\left(G\left(z\right)\right)\right)\right]\right\}$$

where *x* is a real object input in discriminator *D* and *z* denotes the normally distributed latent vectors that represent the random input for generator $G^{\prime }$ as illustrated in Figure 1.

### 3.2. 3D-IWGAN

In the original GAN method, the Kullback–Leibler divergence is minimized when training the networks to generate 3D objects. The drawback of using this method is the slow and unstable training process. A recent study introduced a method called 3D-improved Wasserstein GAN (3D-IWGAN) [25] that attempted to fix this issue and aimed to generate more realistic 3D objects by minimizing the Wasserstein distance between the data and generated distributions. The distance formula is

(2) $$W\left(p_{r},p_{g}\right)=\underset{\gamma ∼\Pi (p_{r},p_{g})}{\inf}E_{(x,y)∼\gamma }[∥x-y∥]$$

where $\Pi (p_{r},p_{g})$ is the set of all possible joint probability distributions between $p_{r}$ and $p_{g}$ and $\gamma \in \Pi (p_{r},p_{g})$ is the set of joint distributions in the continuous probability space. In the definition of Wasserstein distance, the infimum (greatest lower bound) indicates that we are only interested in the smallest cost.

The deviation of the discriminator’s gradients is penalized from unity in 3D-IWGAN. It provides a more essential method for enforcing the Lipschitz constraint. The gradients of a differentiable function are at most one if and only if it is a 1-Lipschitz function. In the formal definition, the loss function for the discriminator in 3D-IWGAN is written as

(3) $$E_{\hat{x}∼p_{g}}\left[D\left(\hat{x}\right)\right]-E_{x∼p_{r}}\left[D\left(x\right)\right]+\lambda E_{\hat{x}∼p_{x}}\left[{\left(∥{\nabla_{\hat{x}}D\left(\hat{x}\right)∥}_{2}-1\right)}^{2}\right]$$

where λ is the gradient penalty, $p_{g}$ is the generator distribution, $p_{r}$ is the target distribution, and $p_{x}$ is the uniform distribution sampling.

The issue of the instability of GAN training can be tackled by employing the IWGAN method. Our system utilized the IWGAN with an enhanced gradient penalty for 3D microstructure reconstruction. In comparison to previous approaches, our system demonstrated more stable results in training of the GAN. Table 2 displays the comparison between our enhanced gradient penalty and other GAN gradient penalties. A detailed explanation of this gradient penalty will be covered in Section 4.

**Figure 2:**
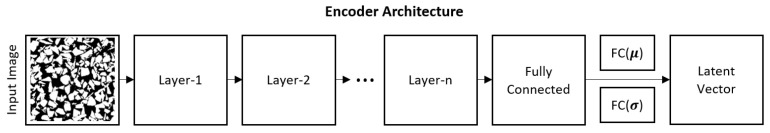
VAE encoder architecture. It encodes data into a lower-dimensional latent space.

A latent vector is needed as an input to the generator network in the 3D-IWGAN system. In a general way, this latent vector is generated randomly using normal or uniform distribution. Meanwhile, our system generates this latent vector using a variational autoencoder (VAE). A VAE [36] consists of two parts: an encoder and a decoder. Its main task in our system is to encode material microstructures into a lower-dimensional latent space and to decode samples from the latent space back into microstructures. A full schematic view of the VAE architecture is given in Figure 2 Our VAE’s decoder network is simultaneously used by the generator network to reproduce the original sample.

## 4. Proposed System

### 4.1. System Architecture

**Figure 3:**
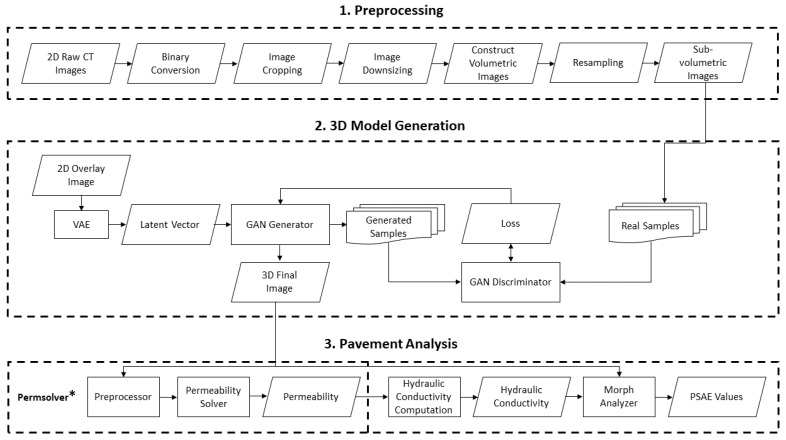
System architecture. This consists of three steps: preprocessing, 3D model generation, and pavement analysis.

The proposed system for the virtual experiment consists of three components: preprocessing, 3D model generator, and pavement analysis. The architecture of the system is illustrated in Figure 3.

In this section, we provide a brief overview of each component of the system. The preprocessing part has several tasks such as cropping, converting, downsizing, and resampling the images. In the model generation part, we use an adversarial network to generate a 3D image from the 2D images of the porous pavement microstructure. This 3D image is used as the input for pavement analysis, which further provides the numerical values of physical properties such as porosity, permeability, and hydraulic conductivity.

### 4.2. Preprocessing

Before implementing the 3D-IWGAN, we need to prepare the image data to be fixed with the requirement of GAN input. The first step of preprocessing is binary image conversion. In this step, the raw CT images of the microstructure are converted into binary images with the dimension of 1130 × 1130 pixels. This binary conversion is based on Otsu’s thresholding method.

**Figure 4:**
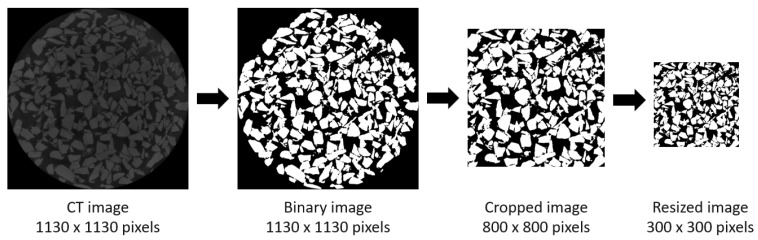
Binary conversion and image cropping. Input data are converted to binary versions, then converted and resized to fit the requirement of system.

In the second step, the image is cropped into a square with the dimension of 800 × 800 pixels, which is the maximum square size inside the circle of the converted binary image. The squared image is then resized into a smaller size of 300 × 300 pixels to fit the system requirement. This is the third step, known as image downsizing. The illustration of binary conversion, image cropping, and downsizing is shown in Figure 4.

**Figure 5:**
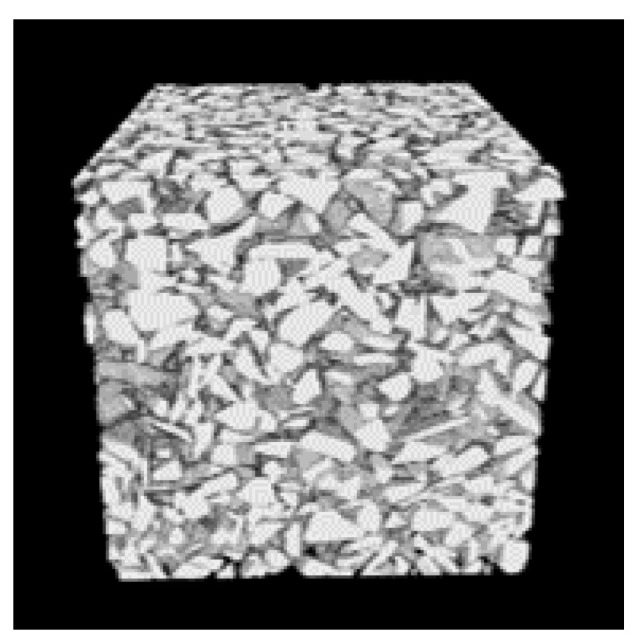
Volumetric image of permeable pavement sample. This consists of 800 images each of 800 × 800 pixel size.

The fourth step of preprocessing consists of volumetric image construction. In this step, a 3D microstructure image is created by stacking the binary images. The stack consists of 800 images of the permeable pavement microstructure and the size of each image is 800 × 800 pixels. The volumetric image of this generated microstructure is shown in Figure 5.

**Figure 6:**
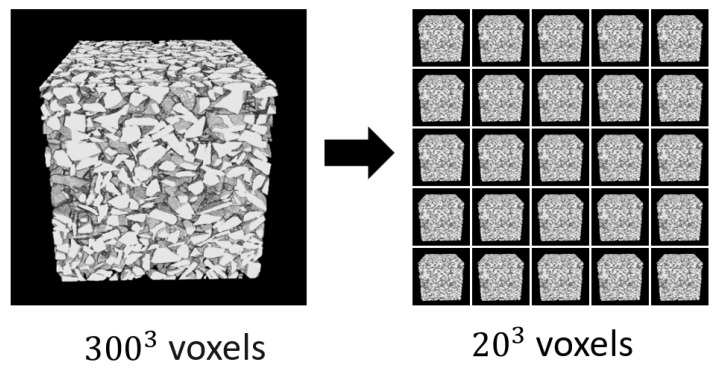
Volumetric image resampling. Overall, 24,389 images of $20^{2}$ voxels are taken from a single $300^{3}$ voxel image.

Image resampling is the fifth step of preprocessing. Since we only have one training image per sample for the generator network, we need to create appropriate images for the training process by extracting sub-volumes from the voxelized binary images. For this experiment, we made approximately 24,389 images of $20^{2}$ voxels from a single sample image of $300^{3}$ voxels, illustrated in Figure 6. These generated images were used as the training images in the generator network.

### 4.3. 3D Model Generation

In this subsection, we shall present details of the 3D model generation including the 3D-IWGAN process and enhanced gradient penalty.

#### 4.3.1. 3D-IWGAN

The 3D model of the pervious pavement microstructure is generated using GAN in the first part of our system (3D model generator). The network takes a 2D image as the input in the generator network. This 2D image is transformed into a 3D image by the deconvolutional neural network. From the generator, the image is delivered to the discriminator network and it is tested against the real sample image of the microstructure to check whether the generated 3D model is real or fake using the discriminator.

**Figure 7:**
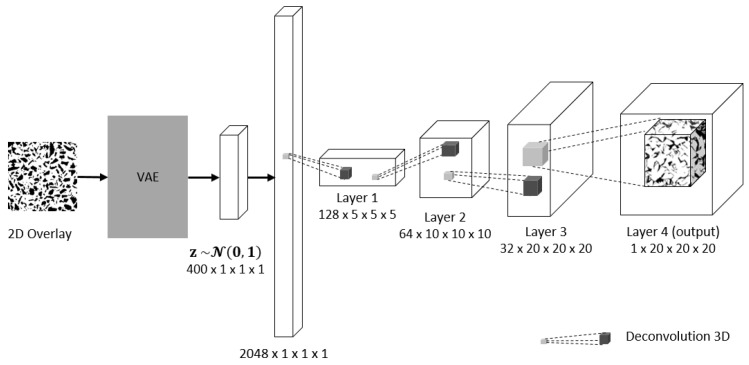
Generator network. It takes a fixed-length 400-dimensional latent vector as the input and generates a sample in the domain.

The 3D-IWGAN network architecture used for 3D microstructure reconstruction in module one consists of two independent networks, the generator *G* and discriminator *D*. A 400-dimensional latent vector with normal distribution serves as the input for the generator network. Figure 7 shows the architecture of the generator network.

**Figure 8:**
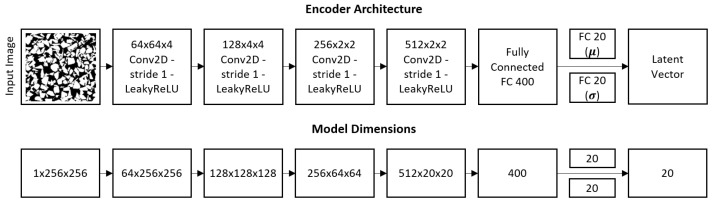
VAE architecture.

The latent vector was generated using the VAE, whose architecture is identical to that of discriminator network in the 3D image generation of GAN. Figure 8 illustrates the architecture of the VAE. This input passes through a fully connected layer with 2048 nodes. After getting through the fully connected layer, it passes through four deconvolutional layers whose length of stride and kernel size are 1 and 5, respectively. The first three deconvolutional layers in the generator network have a batch normalization layer and use the rectified linear unit (ReLU) as their activation function to address the vanishing gradient problem and promote sparse activations, while the fourth layer uses a hyperbolic tangent (tanh) as its activation function to make sure that our generating samples are in the range [−1, 1]. The output of the generator network is a 3D image of size 20 × 20 × 20 voxels.

The discriminator network D takes an input image of size 20 × 20 × 20 voxels. This input passes through four 3D convolutional layers, followed by a final fully connected layer. It is then condensed to a single value known as the discriminator output. The features of the architecture of the generator and discriminator networks are listed in Table 3 and Table 4, respectively.

#### 4.3.2. Enhanced Gradient Penalty

Various types of gradient penalty are used to improve the convergence and stability of GAN training. One of the most commonly used gradient penalties is the one-centered gradient penalty [24], which is described as

(4) $$E_{\hat{x}∼p_{g}}\left[D\left(\hat{x}\right)\right]-E_{x∼p_{r}}\left[D\left(x\right)\right]+\lambda E_{\hat{x}∼p_{x}}\left[{\left(∥{\nabla_{\hat{x}}D(\hat{x})∥}_{2}\right)}^{2}\right]$$

In this paper, we used an enhanced gradient penalty for our 3D-IWGAN system. The original 3D-IWGAN gradient penalty is being modified to enhance the training stability of our GAN. Its formal definition is as follows:

(5) $$\lambda E_{\hat{x}∼p_{x}}\left[{\left(∥{\nabla_{\hat{x}}D(\hat{x})∥}_{2}-1\right)}^{2}\right]$$

The gradient penalty used in this study is a form of zero-centered gradient penalty. In the zero-centered gradient penalty, we want to set the gradient to zero as the generator distribution $\left(p_{g}\right)$ approaches the target distribution $\left(p_{r}\right)$. When $p_{g}=p_{r}$, the gradient in connection to all datapoints on the line segment between a pair of real and fake samples should be zero.

### 4.4. Pavement Analysis

In the final part of our system, we analyzed the physical properties of the microstructure, such as porosity and permeability, using the 3D model output from the model generator part. The pavement analysis comprised two sub-parts: (1) *PermSolver* and (2) *MorphAnalyzer*.

*PermSolver* is an open-source framework developed by NIST [37]. It takes a 3D image of the microstructure as the input, converts it into x-y-z velocity and pressure components, then solves the Stokes equation using a finite difference scheme. By solving this equation, we can get the computed permeability of the porous microstructure.

Other than permeability values, we can also calculate the hydraulic conductivity (*K*) with this equation:

(6) $$K=k\frac{\rho g}{\mu }$$

where *k* is the permeability value (from *Permsolver*), ρ is the density of fluid, *g* is the acceleration due to gravity, and μ is the dynamic viscosity of the fluid. In the following Section 5 the results of computing the hydraulic conductivity are described.

*MorphAnalyzer* is a tool that is used to compute the important characteristics of microstructure morphology. In our model we use three characteristics: porosity, specific area, and Euler characteristic. Porosity is calculated using the total number of pores based on the output of the 3D model reconstruction module. The specific area and Euler characteristic are obtained by using *ImageJ* [38]. The values extracted in this part are important in the investigation of the adsorption process and pore network connectivity of the permeable pavement microstructure.

## 5. Implementation and Evaluation

In this section, we present the performance evaluation of our system.

### 5.1. Experimental Setup

#### 5.1.1. Hardware and Software

To evaluate the proposed system, we used one commodity machine. This machine is equipped with an Intel Xeon(R) CPU E5-2609 v4 @ 1.70 GHz x 16, with 64 GB memory, NVIDIA GeForce GTX 1060 6 GB, and runs on a 64-bit Ubuntu 16.04 operating system.

We implemented our approach in Python 2.7 using Tensorflow-GPU 1.2.1 performed with CUDA Toolkit 8.0 and cuDNN 6.0.

#### 5.1.2. Dataset

The dataset used for this work was extracted from nine different samples of permeable pavement. The sample image was scanned using a CT machine with a voxel size of 88.194 μm. The size of one sample image after binary conversion to 8-bit resolution was $300^{3}$ voxels. We resampled this image to 24,389 images with a size of $20^{3}$ voxels. The size of this dataset was approximately 14.4 GB.

### 5.2. Experimental Results

#### 5.2.1. 3D Pavement Reconstruction

The reconstruction process started by defining some hyperparameters for the generator and discriminator networks. We set $10^{-4}$ as the generator learning rate. Thus, it learns every 5 batches with batch normalization in all layers except the final layer. For the discriminator network, the learning rate is set to the same value as the generator network but it learns on each batch. The stochastic gradient descent using the Adam optimizer was used to perform the learning process, with momentum $\beta_{1}$ = 0.5 and $\beta_{2}$ = 0.9 for both networks.

**Figure 9:**
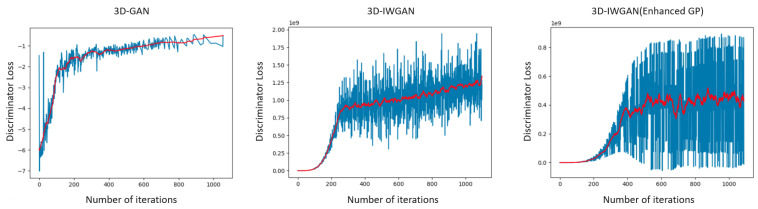
Discriminator loss comparison. The convergence and quality of generated objects are tracked by the calculated loss.

We trained the network and calculated the loss for the discriminator network. The calculated loss is used to track the convergence and quality of generated objects, depicted in Figure 9. The first plot on the left is the GAN discriminator loss, the second plot in the center is the calculated loss for the original 3D-IWGAN, while the third plot is that of the 3D-IWGAN (with enhanced gradient penalty) discriminator loss. From the plots, we can see that the discriminator loss of 3D-IWGAN (enhanced GP) has a bouncing value in the beginning but it has relatively stable values after 600 iterations. Meanwhile, the discriminator loss of GAN and original GAN increase until 1000 iterations. Although our result shows that this value does not converge to zero, it is more stable in comparison to other methods. The stability in GAN’s loss is also important to prevent the collapse and failure modes. To check the accuracy of our model, we added the computation of physical properties to display the similarities between our generated 3D model and the real sample. The result of this computation is presented in Permeable Pavement Analysis, Section 5.2.2.

**Figure 10:**
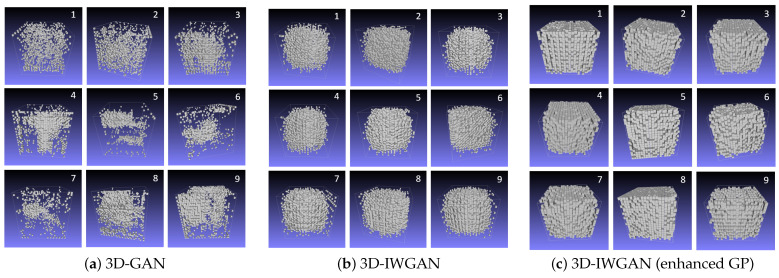
Comparison of voxelized 3D Images. Images are reconstructed by GAN, 3D-IWGAN, and 3D-IWGAN (enhanced GP) methods.

The result of the 3D reconstructed image using GAN is displayed in Figure 10a. Figure 10b illustrates the image generated using the original 3D-IWGAN, and the image generated using 3D-IWGAN (enhanced GP) is presented in Figure 10c. The images generated using the GAN method have more pores in comparison to those generated using the original 3D-IWGAN and our 3D-IWGAN, while the images generated by the original IWGAN have more solid phases in comparison to other methods. Among the three methods, the 3D-IWGAN (enhanced GP) shows more realistic images of the pavement microstructure.

**Figure 11:**
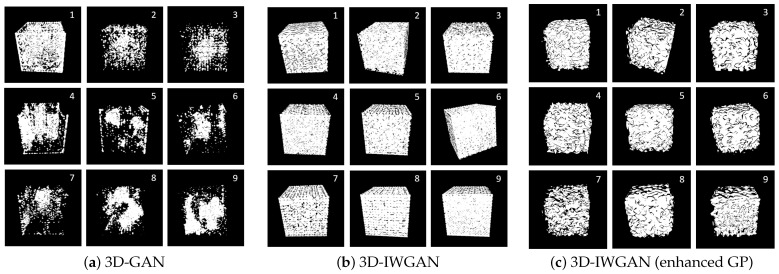
Comparison of generated 3D images.

We reconstructed the microstructure for nine different samples, whose results are presented in Figure 11.

#### 5.2.2. Permeable Pavement Analysis

The final part of our system is pavement analysis. After obtaining the 3D image of the pavement microstructure, we attempted to analyze the physical properties of the generated images. We attempted to evaluate five properties: porosity, permeability, hydraulic conductivity, specific surface area, and Euler characteristic.

**Figure 12:**
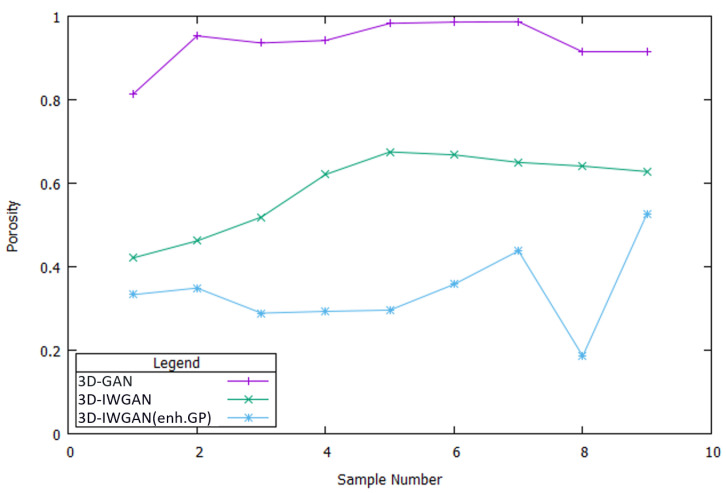
Computed porosity of 9 different samples. Our proposed method shows the highest result for the value of porosity among other methods.

In porous media such as permeable pavement, flow properties can be related to porosity, which is required to determine other properties such as permeability and hydraulic conductivity. Therefore, it needs to be estimated first. According to the ASTM standard, the porosity for permeable pavement is approximately 15–35%. Figure 12 shows the calculated porosity for the nine images generated using the IWGAN method. In comparison to the result of the previous GAN model, the proposed model shows better results for the values of porosity. The calculation results of the porosity from our generated 3D images shows that 66.67% of our samples are in the correct range of the standard value. Our method is better than the other methods, including none of the samples that fall within the appropriate range of standard values. The porosity values of the generated images from 3D-IWGAN (enhanced GP) illustrate a 66.67% improvement over the previous methods (3D-GAN and 3D-IWGAN).

We also calculated other properties such as the specific surface area and Euler number. The specific surface area is used to define the adsorption and dissolution processes in porous media, while the Euler number is used to characterize the connectivity of porous media. This is an important factor that determines the ability of fluids to flow. A less negative Euler number indicates a reduction in pore network connectivity. The values of surface area and Euler number are presented in Table 5. According to the ASTM standard, permeable pavement has a value of surface area approximately in the range 5–15%. Our results demonstrate that all samples are in the correct range (5–15)% of the surface area.

**Figure 13:**
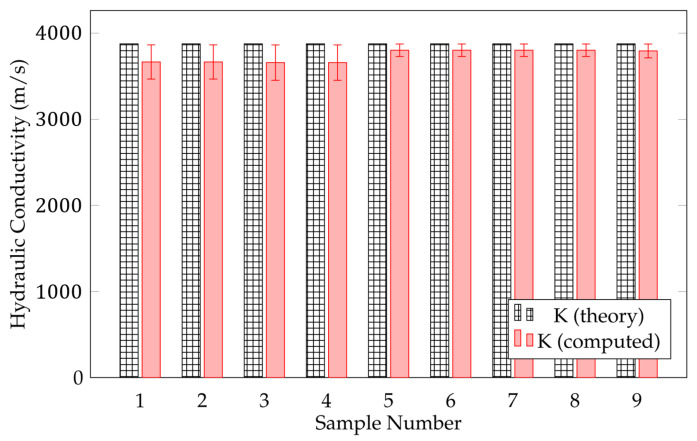
Computed hydraulic conductivity. The errors range between 1.9% and 5.6% among nine different samples.

After measuring the morphological values, we tackled the final part in our system: computing the permeability and hydraulic conductivity for each sample. The results of this computation can be used to determine the clogging potential of permeable pavement. The result for hydraulic conductivity is illustrated in Figure 13. It shows that the error difference in the nine different samples ranges between 1.9% and 5.6% or an average of approximately 3.54%.

The results of pavement analysis demonstrate that the proposed 3D model yielded better values than the model generated using 3D-GAN and original 3D-IWGAN. From the nine different samples, the average error difference in hydraulic conductivity computation was found to be below 5%. Moreover, the values of physical properties extracted through the proposed 3D model using our GAN method are closer to the baseline (theory) values than other GAN methods.

### 5.3. Discussion

We summarize our two major finding based on our experimental results. First, our framework can successfully generate 3D images of porous pavement microstructure from a single 2D image using 3D-IWGAN with enhanced GP. Figure 10 and Figure 11 illustrate the generated 3D microstructure images as a visualization. Second, the generated 3D images are realistic in terms of the physical properties of permeable pavement extracted from our generated images. From Figure 12 and Table 5, we observe that the computed values of porosity and surface area fall in the correct ranges of the standard values. Figure 13 also displays a low error value of about 3.54% on average in nine different samples.

There are some limitations that restrain us from obtaining 100% accuracy despite getting decent results in 3D image generation and the computations of physical properties. Some limitations arise from the limited 2D image dataset as the input whereas others arise from the computational resources for our proposed framework. To provided a 2D input image for our framework, scanned images of pavement microstructure are needed at the pre-processing step. Since we only had a limited set of scanned images, we applied image resampling to obtain enough 2D images for our experiments. Powerful computational resources are important for generating 3D images with high resolution. For future experiments, several machines can be utilized as a distributed way to obtain large 3D images with higher resolutions.

## 6. Conclusions

Although permeable pavement has been extensively used to allow rainwater to pass through the pores into the ground, to control water quality, and to reduce the surface temperature, it has some obstacles during utilization: obtaining samples, maintenance cost, and clogging issues.

To overcome above-mentioned problems, in this paper, we proposed a three-dimensional microstructure reconstruction framework for permeable pavement analysis as one of the virtual experiment solutions. Since we utilized the generative model of 3D-IWGAN with an enhanced gradient penalty, our framework can effectively generate 3D microstructure reconstruction from a single 2D image of permeable pavement. To the best of our knowledge, 3D-IWGAN has not been used for the 3D model reconstruction of permeable pavement materials. From the visualization of the generated 3D microstructure images from nine samples, we have observed that our framework generates realistic 3D images.

When analyzing the flow in porous media, it is crucial to improve the values of porosity. Incorrect values of porosity will affect the other physical properties of permeable pavements such as permeability and hydraulic conductivity. These two properties are also important to assess the quality of generated images that are later used for pore network analysis. We have demonstrated that our framework generates more realistic 3D microstructure images, maintaining the values of these physical properties within the range of the standard values. In particular, the porosity values of the generated 3D images from 3D-IWGAN with enhanced GP were improved by 66.67% compared to the previous methods (3D-GAN and 3D-IWGAN).

Our proposed 3D microstructure reconstruction framework can be extended in two directions. First, we can add an additional module that generates and analyzes the pore network to conduct comprehensive virtual experiments. Second, we can combine some empirical methods with our framework to increase the quality of the generative model and avoid random errors.

## Figures and Tables

**Table 1:** 2D/3D object reconstruction methods with statistical and deep learning approaches.

Approach	Method	Application	Output
Statistics	Two-point Correlation	geometry reconstruction, analysis	2D/3D microstructure
Deep Learning	CDBN	2D microstructure reconstruction	2D microstructure
Deep Learning	3DGAN	3D object reconstruction	3D objects (non-microstructure)
Deep Learning	DCGAN	2D object reconstruction	2D microstructure
Deep Learning	IWGAN	2D/3D object reconstruction	2D/3D objects (non-microstructure)
Deep Learning	3D-IWGAN (enhanced GP)	3D microstructure reconstruction	3D microstructure

**Table 2:** Gradient penalty comparison of different GAN methods. This measures the squared difference between the norm of the gradient of the predictions with respect to the input images and 1 in all cases.

Method	Gradient Penalty	Objective Function
3DGAN	none	$E_{x∼p_{data}}\left[logD\left(x\right)\right]+E_{z∼p_{noise}}\left[log\left(1-D\left(G\left(z\right)\right)\right)\right]$
3D-IWGAN (1-GP)	$\lambda E_{\hat{x}∼p_{x}}\left[{\left(∥{\nabla_{\hat{x}}D(\hat{x})∥}_{2}-1\right)}^{2}\right]$	$E_{\hat{x}∼p_{g}}\left[D\left(\hat{x}\right)\right]-E_{x∼p_{r}}\left[D\left(x\right)\right]+\lambda E_{\hat{x}∼p_{x}}\left[{\left(∥{\nabla_{\hat{x}}D(\hat{x})∥}_{2}-1\right)}^{2}\right]$
3D-IWGAN (enhanced-GP)	$\lambda E_{\hat{x}∼p_{x}}\left[{\left(∥{\nabla_{\hat{x}}D(\hat{x})∥}_{2}\right)}^{2}\right]$	$E_{\hat{x}∼p_{g}}\left[D\left(\hat{x}\right)\right]-E_{x∼p_{r}}\left[D\left(x\right)\right]+\lambda E_{\hat{x}∼p_{x}}\left[{\left(∥{\nabla_{\hat{x}}D(\hat{x})∥}_{2}\right)}^{2}\right]$

**Table 3:** Generator network architecture. The first three layers use ReLU activation functions and the last layer uses the Tanh activation function.

Layer	Type	Filters	Stride	Padding	Batchnorm	Activation Function
1	Deconvolution 3D	128	1	0	Yes	ReLU
2	Deconvolution 3D	64	2	1	Yes	ReLU
3	Deconvolution 3D	20	2	1	Yes	ReLU
4	Deconvolution 3D	1	1	1	No	Tanh

**Table 4:** Discriminator network architecture. All layers use the LeakyReLU activation function and no batch normalization is used.

Layer	Type	Filters	Stride	Padding	Batchnorm	Activation Function
1	Convolution 3D	1	1	1	No	LeakyReLU
2	Convolution 3D	32	2	1	No	LeakyReLU
3	Convolution 3D	64	2	1	No	LeakyReLU
4	Convolution 3D	128	1	0	No	LeakyReLU

**Table 5:** Computed surface area and Euler number of 9 samples.

Sample	Specific Surface Area	Euler Number
1	7.37%	−1.27 ×$10^{3}$
2	8.93%	−4.53 ×$10^{2}$
3	9.45%	−3.21 ×$10^{2}$
4	8.77%	−5.25 ×$10^{2}$
5	10.93%	−2.19 ×$10^{2}$
6	8.65%	−4.16 ×$10^{2}$
7	7.91%	−5.98 ×$10^{2}$
8	14.26%	−1.09 ×$10^{2}$
9	6.17%	−6.26 ×$10^{2}$
